# Animal-Borne Acoustic Transceivers Reveal Patterns of at-Sea Associations in an Upper-Trophic Level Predator

**DOI:** 10.1371/journal.pone.0048962

**Published:** 2012-11-14

**Authors:** Damian C. Lidgard, W. Don Bowen, Ian D. Jonsen, Sara J. Iverson

**Affiliations:** 1 Department of Biology, Dalhousie University, Halifax, Nova Scotia, Canada; 2 Population Ecology Division, Bedford Institute of Oceanography, Department of Fisheries and Oceans, Dartmouth, Nova Scotia, Canada; Hawaii Pacific University, United States of America

## Abstract

Satellite telemetry data have substantially increased our understanding of habitat use and foraging behaviour of upper-trophic marine predators, but fall short of providing an understanding of their social behaviour. We sought to determine whether novel acoustic and archival GPS data could be used to examine at-sea associations among grey seals (*Halichoerus grypus*) during the fall foraging period. Fifteen grey seals from Sable Island, Canada were deployed with Vemco Mobile Transceivers and Satellite-GPS transmitters in October 2009, 13 of which were recaptured and units retrieved 79±2.3 days later during the following breeding season, December 2009–January 2010. An association between two individuals was defined as a cluster of acoustic detections where the time between detections was <30 min. Bathymetry, travel rate, and behavioural state (slow and fast movement) were determined for each GPS archival point (3.7±0.1 locations recorded per hour). Behavioural state was estimated using a hidden Markov model. All seals had been involved in associations with other instrumented seals while at sea, with a total of 1,872 acoustic detections recorded in 201 associations. The median number of detections per association was 3 (range: 1–151) and the median duration of an association was 0.17 h (range: <0.1–11.3 h). Linear mixed-effects models showed that associations occurred when seals were exhibiting slow movement (0.24±0.01 ms^−1^) on shallow (53.4±3.7 m) offshore banks where dominant prey is known to occur. These results suggest the occurrence of short-term associations among multiple individuals at foraging grounds and provide new insights into the foraging ecology of this upper-trophic marine predator.

## Introduction

The social organization and mating systems of pinnipeds have been well studied for a number of species. This is due, in part, to their dependence on a solid substrate for parturition (i.e., land or ice), allowing individuals to be easily observed [1], [2]. In contrast our understanding of sociality of pinnipeds during the non-breeding season is less well known for the simple reason that the marine environment makes behavioural observation difficult. Satellite telemetry data have substantially increased our understanding of the at-sea movements of pinnipeds and other marine predators [3], [4], [5] but these data fall short of providing information on the nature of associations among individuals. This is because of the imprecision attached to geographical locations, and the irregular timing and low frequency of the data, although considerable efforts have been made to overcome these issues [6].

In the absence of information to the contrary, it has been generally assumed that most pinnipeds do not associate with one another while at sea, but rather travel and forage independently. However, a recent study on the movement characteristics of grey seals (*Halichoerus grypus*) has shown that naïve young-of-the-year, who would be expected to have poor knowledge of their marine environment, move in a pattern that is not significantly different to that of sub-adults and adults, suggesting they may be associating with older individuals while at sea [7]. Similar results have been found for the southern elephant seal (*Mirounga leonina*) [8]. In cattle, naïve animals have been shown to be more efficient at finding preferred food resources when in the presence of an experienced animal [9], [10]. Predators should attempt to maximise their energy intake and this can only be achieved through having some knowledge of the resource distribution in their environment. Given that foraging patches in a marine environment vary in quality, both spatially and temporally, it would be beneficial for individuals to learn the location of high quality patches through travelling with other individuals [11]. The occurrence of multiple individuals in the same space at the same time would also allow for the possibility that the behaviour of one or more individuals may influence the presence or behaviour of other individuals and thus provide the foundation for a social structure [12] that may play an important role in foraging behaviour. Aggregative foraging behaviour may also have important consequences for spatial population dynamics of prey species and thus ecosystem function [13].

The recent development of a novel acoustic technology [14], [15], [16], in combination with GPS data, may provide researchers with the ability to examine at-sea associations among individuals in species that are otherwise inaccessible. The Vemco Mobile Transceiver (VMT; www.vemco.com) combines archival and acoustic tag technology. The VMT houses a miniaturised acoustic transceiver that records unique acoustic codes from other tagged individuals (within approximately 300 m) and transmits its own unique code. Thus, when coupled with GPS data, time-stamped and geo-referenced records of proximity can be collected over extended periods of time. The VMT has been deployed successfully on Galapagos sharks (*Carcharhinus galapagensis*) and northern elephant seals (*M. angustirostris*) as pilot studies determining the feasibility of using this technology to examine inter- and intra-animal encounters [14], [15]. Although both studies demonstrate proof of concept, they fall short of being able to address relevant biological questions due mainly to small sample sizes. In the current study, we used this novel acoustic technology to examine intra-species encounters in a marine upper-trophic predator, the grey seal.

The grey seal is a size-dimorphic phocid that inhabits temperate waters on both sides of the North Atlantic Ocean. In the northwestern Atlantic, the grey seal has a broad continental shelf distribution from the Gulf of Maine north to the Gulf of St. Lawrence with the largest breeding colony on Sable Island [3], [4], [17], [18]. Grey seals from Sable Island forage at ‘hot-spots’ on shallow offshore banks on the Eastern Scotian Shelf [4] where their main prey item, the sand lance (*Ammodytes dubius*) is known to occur [19], [20], [21]. It is thus possible that interactions among individuals may occur at sea due to the sharing of a common resource or due to the occurrence of stable associations that persist beyond the foraging grounds.

Upper-trophic marine predators, such as the grey seal, can have top-down effects on marine ecosystems [22] and commercial fish stocks [23]. If grey seals are foraging co-operatively at these ‘hot-spots’, this study may help to elucidate this behaviour and the potential impact an aggregative response may have on their prey, contributing toward geographical variation in the depletion of food resources. For example, two or more individuals foraging as a group may have a greater impact on their prey than the same individuals foraging alone.

In this study, we sought to determine whether the VMT combined with Fastloc™ GPS telemetry could be used to examine at-sea associations among grey seals during the non-breeding period, a time when they are mainly foraging. We also examined the spatial and temporal pattern of associations to provide preliminary insight into the nature of these associations.

## Materials and Methods

### Ethics Statement

This research was conducted in accordance with guidelines for the use of animals in research [24] and of the Canadian Council on Animal Care. The research protocol for this study was approved by the University Committee on Laboratory Animals, Dalhousie University's animal ethics committee (animal care protocol: 08-088) and Department of Fisheries and Oceans, Canada (animal care permit and license: 08-16; M-09-40).

### Study Site, Study Animals, and Attachment and Use of Telemetry Instruments

**Figure 1:**
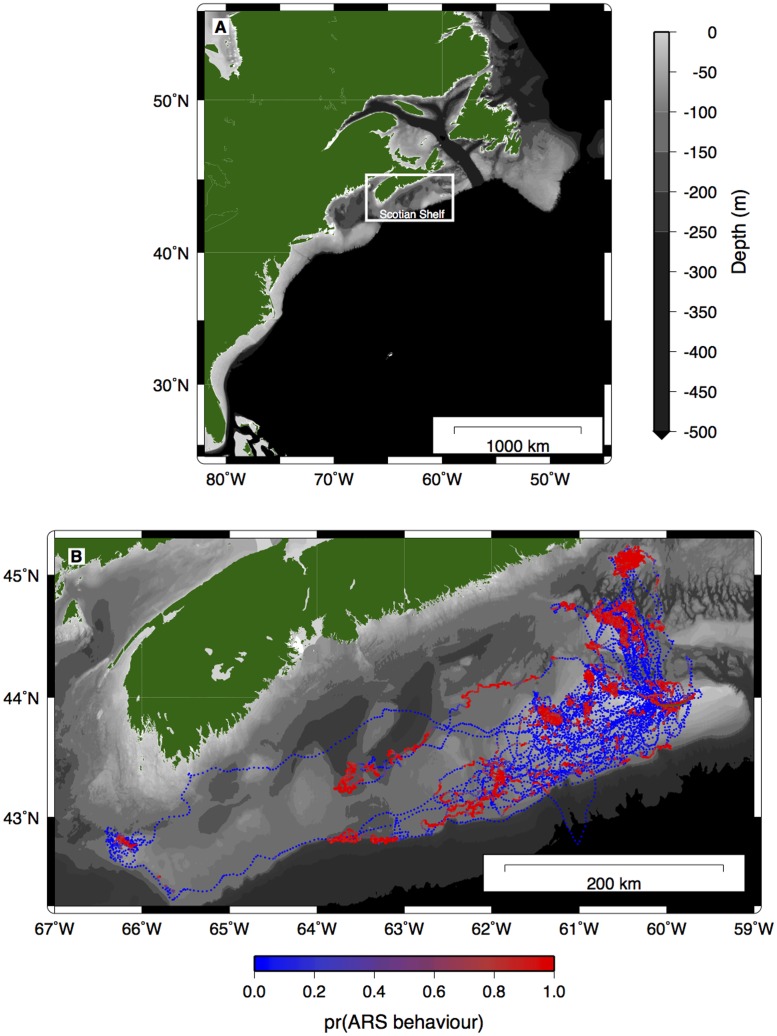
A) Study area and B) GPS movement tracks for the 13 grey seals studied. Pr(ARS behaviour) is a continuous measure (0 to 1) of the probability of exhibiting area- restrictive search behaviour according to the hidden Markov model.

The study was conducted between 15^th^ October 2009 and 7^th^ January 2010 on Sable Island (43°55′N, 60°00′W) and the Eastern Scotian Shelf, northwest Atlantic Ocean (Fig. 1A). The Eastern Scotian Shelf is a large geographical area (∼108,000 km^2^) composed of a series of offshore shallow banks and inshore basins separated by deep gullies and canyons [25], and is an important foraging area for the grey seal [26].

During the period 1969 and 2002, a sample of males and females was branded at weaning providing a pool of individually identifiable, known-age adults. Fifteen of these known-age adults (11 to 24 years) were captured between 15 and 20 October 2009. The distance between consecutive captures was 8.4±10.7 km (0.4–30.8 km). At each capture, individuals were weighed using a 300 kg (±1 kg) Salter spring balance. Individuals were then immobilized with an intra-muscular injection of the chemical anaesthetic Telazol (males 0.45 mg kg^−1^; females 0.90 mg kg^−1^) to allow attachment of telemetry and data-logging devices and to obtain an accurate measure of standard dorsal length [27]. Each seal was fitted with a VHF transmitter (164–165 MHz, Advanced Telemetry Systems, www.atstrack.com), Mk10-AF Fastloc™ GPS tag (Wildlife Computers, www.wildlifecomputers.com) and a Vemco Mobile Transceiver (VMT) tag (Vemco, www.vemco.com). The VHF tag was used to locate animals returning to Sable Island during the breeding season. The MK10-AF tag was programmed to transmit ARGOS and GPS data and to archive GPS data that were downloaded on recovery of the tag. As GPS tags are relatively new, we tested two GPS sampling protocols with respect to their impact on battery life and thus the duration of data collection. Four units were programmed to record a GPS location every five minutes (maximum of 48 failed attempts per hour and unlimited GPS attempts per day) and 11 units recorded a GPS location every 15 minutes (maximum of 16 failed attempts per hour and unlimited GPS attempts per day). GPS attempts were suspended when the unit was dry >20 min and a location had been attained. The VMT is a 69 kHz coded transceiver that alternates between transmitting an acoustic code (unique series of acoustic pings) and listening for codes transmitted from other Vemco 69 kHz coded transmitters. Acoustic codes are unique due to the time interval between pings and the length of time it takes to transmit the full code. The VMT was programmed to transmit on an irregular schedule (to avoid synchronised transmissions among VMTs), every 60 to 180 s, to blank the receiver for 260 ms at each transmission (to prevent the tag from receiving its own transmission), and remain in listening mode for the remainder of time. Peak sensitivities for hearing in phocids is between 10 and 30 kHz with a high frequency limit of ∼60 kHz [28], thus we did not expect these units to interfere with the behaviour of the animal.

The VHF transmitter was attached to the MK10-AF unit using a stainless steel hose clamp and the whole unit was attached to the fur on the top of the head using a five-min epoxy [29]. The VMT was attached in the same manner as for the MK10-AF, but was located on the back toward the rear of the animal to maximise the likelihood that the tag remained underwater when the animal was at the surface and to minimise electrical interference with the MK10-AF. The tag mass burden was 0.25% for males and 0.28% for females. Individuals were recaptured during the subsequent breeding season (December 2009 to January 2010) to determine final body mass and recover instruments.

### Data Analysis

Although Argos and GPS locations were available from the data transmitted to Service Argos, the archival GPS data provided more locations and of higher accuracy. Therefore, we report only those data here. To determine GPS locations, archival GPS data from each MK10-AF were analyzed using propriety software from the manufacturer (WC-DAP; www.wildlifecomputers.com) and archival ephemeris data (www.cddis.gsfc.nasa.gov). Locations acquired from <5 satellites and/or with a residual error >30 were of lower accuracy [30], [31] and removed from the dataset. VMT detections comprised a date-time stamp and the identities of the VMTs detected, these were downloaded and visualised using the dedicated software VUE (www.vemco.com).

False detections of other VMTs may occur for a variety of reasons such as the production of legal codes from the arrival of multiple codes from other active transmitters at the receiver at the same time. False detections (*n* = 26) were identified using proprietary software (Vemco) and subsequently removed from the dataset. In the majority of cases (97%), true detections between two transceivers comprised a pair of detections, one from the transmitting transceiver and another from the receiving transceiver. Thus, to determine the number of detections logged between two transceivers only one of the possible two detections was counted. Multiple detections are expected when two individual seals associate with each other. Therefore, it was necessary to operationally define when one association ended and another began. Visual inspection of a histogram displaying times between detections <200 min, (Fig. S1, see supplementary material), indicated that a gap of >30 min between detections could be considered to constitute the end of an association. For associations that involved only a single detection (*n* = 53) the duration of the association was set at three minutes since after this time, based on the transmission rate of the VMT, another detection would have occurred if the two individuals were still together. Using the date-time data from each VMT, the location of each seal-seal association was estimated using linear interpolation between GPS locations.

To assign bathymetric values and calculate travel-rate, each seal track was divided into a series of 60 min time-steps (24 time-steps day ^−1^) with GPS data averaged within each step. Bathymetry (as a proxy for seal habitat) and travel rate were determined for each step. Although it was possible for a single association to span two or more time steps, we were only interested in whether an association occurred or not during each step. To examine whether seal-seal associations preferentially occurred at certain depths, we sampled bottom depths from randomly chosen locations within the region of the Scotian Shelf traversed by each of the 13 seals. Using kde2d from the MASS R package [32], we fit 2D kernel density estimators over a 500×220 grid to each seal track and used the resulting probability densities as weights to define the intensity of sampling in 2D space. Locations were sampled from the grid in direct proportion to the number of locations per each seal track. This approach yielded a scheme that sampled regions with a high density of seal locations more intensively than other regions (Fig. S2, see supplementary material), it also accounted for differences in habitat use amongst individual seals. Bathymetric values associated with these sampled locations were obtained from the Canadian Hydrographic Service (www.dfo-mpo.gc.ca) bathymetry data with a 0.25 min resolution. We also examined bathymetric and travel rate distributions along the seal tracks conditional upon association state. We used generalized linear mixed models [32], [33], fit using glmmPQL from the MASS R package, to estimate the differences in bathymetry and travel rate conditional on association state of the seals. We treated the seals as a random effect and accounted for the serial dependence of observations within seal tracks using a first-order autoregressive correlation structure.

To determine whether the spatial distribution of presumed seal foraging activity was correlated with seal associations, we used a hidden Markov model (HMM; [34]). The HMM provides an objective way to discriminate between hidden movement states and thus is advantageous over simple visual inspection of data. Further, given that location data in this study were based on GPS that has inherently low positional error [31], [35], this makes the HMM appropriate for these data as there is no need to account for measurement error. In the present context, the HMM is a state-space model that assumes seal travel rate is conditional upon two discrete, unobserved movement states: fast and slow movement, where slow movement (probability of Area Restricted Search, p(ARS) >0.5) is assumed to be associated with area restricted search, active foraging, and/or resting behaviours [36]. We used the approach of [37] for modelling the travel rate data, assuming the travel rates (move distance over 1 hr periods) arise from two different exponential distributions:
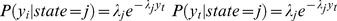
where *y_t_* are the travel rate observations, *λ_j_* is the average distance moved during a 1-hr period, and *j* indexes the movement state (fast or slow movement). See [37] for further details.

Statistical analyses and generation of plots were conducted within R 2.14.1 [32]. All maps were generated using the Generic Mapping Tools [38]. Standard error is reported as the measure of variability.

## Results

Of the 15 seals studied, 13 (eight females and five males) were re-captured between 31 December 2009 and 7 January 2010 and their instruments removed. Two males did not return to Sable Island. One spent the entire breeding season off Brown's Bank south of Sable Island, and data transmission from the other was lost on 20 November 2009, several weeks before the start of the breeding season. Data from the 13 recovered VMTs included detections from the male that moved to Brown's Bank and these data were included in the analysis. The average deployment period was 79±2.3 days (*n* = 13). The mean number of archival GPS locations per hour was 3.7±0.1.

**Figure 2:**
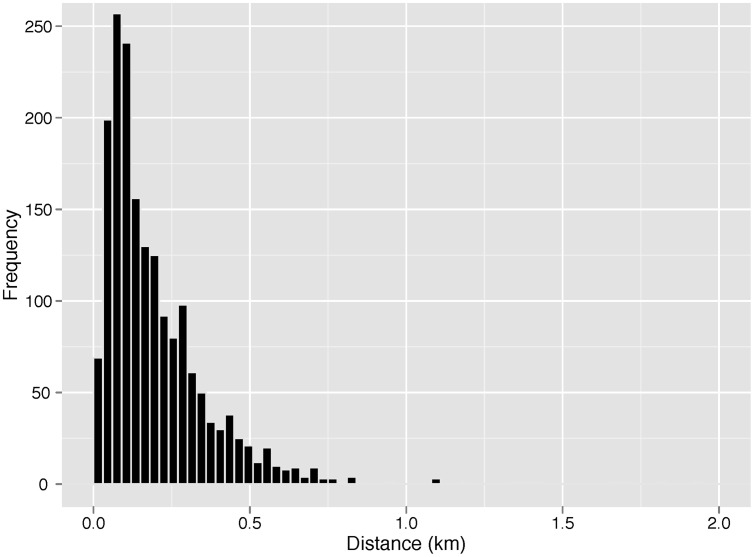
A histogram showing the distances (km) between detections for grey seals (N = 13) on the Eastern Scotian Shelf.

All of the 13 recovered VMTs recorded transmissions from at least one of the 14 seals studied. After false detections (*n* = 26) were removed, 1,892 true detections remained. A histogram of the distances between detections (derived from GPS locations, Fig. 2) shows that the majority fell within 0.05–0.2 km (median = 0.15 km, Q_25_–Q_75_ = 0.08–2.7 km). Using our operational definition of an association, the true detections occurred in 201 seal-seal associations (Table 1). The number of detections per association varied widely from 1 to 151 (median = 3, Q_25_–Q_75_ = 1–9). The duration of seal-seal associations was also highly variable ranging from a single detection to a series of detections over an 11.3 h period (median = 0.17 h, Q_25_–Q_75_ = 0–0.62 h). There were significantly more associations between males (94; 46.8%) than between a male and female (52; 25.9%) or between females (55; 27.4%; G_adj_ = 7.79, df = 2, *p*<0.05).

**Table 1:** Number of associations (detections) between adult grey seals (*n* = 14) deployed on Sable Island for the period 15^th^ October 2009 to 7^th^ January 2010.

	98427	98428	98429	98430	98431	98432*	98433	98434	98435	98436	98437	98438	98439	98440
98427		3 (24)								3 (28)				
98428					15 (124)					2 (12)				
98429									1 (13)	1 (1)				
98430						2 (4)		12 (59)			7 (32)		11 (102)	
98431										2 (6)				
98432*								13 (31)			13 (34)		11 (18)	1 (1)
98433								2 (7)				1 (3)	4 (18)	
98434											13 (44)	1 (7)	6 (28)	
98435											12 (104)			
98436														
98437													12 (112)	
98438													17 (339)	19 (377)
98439														16 (363)
98440														

• Telemetry instruments not recovered.

**Figure 3:**
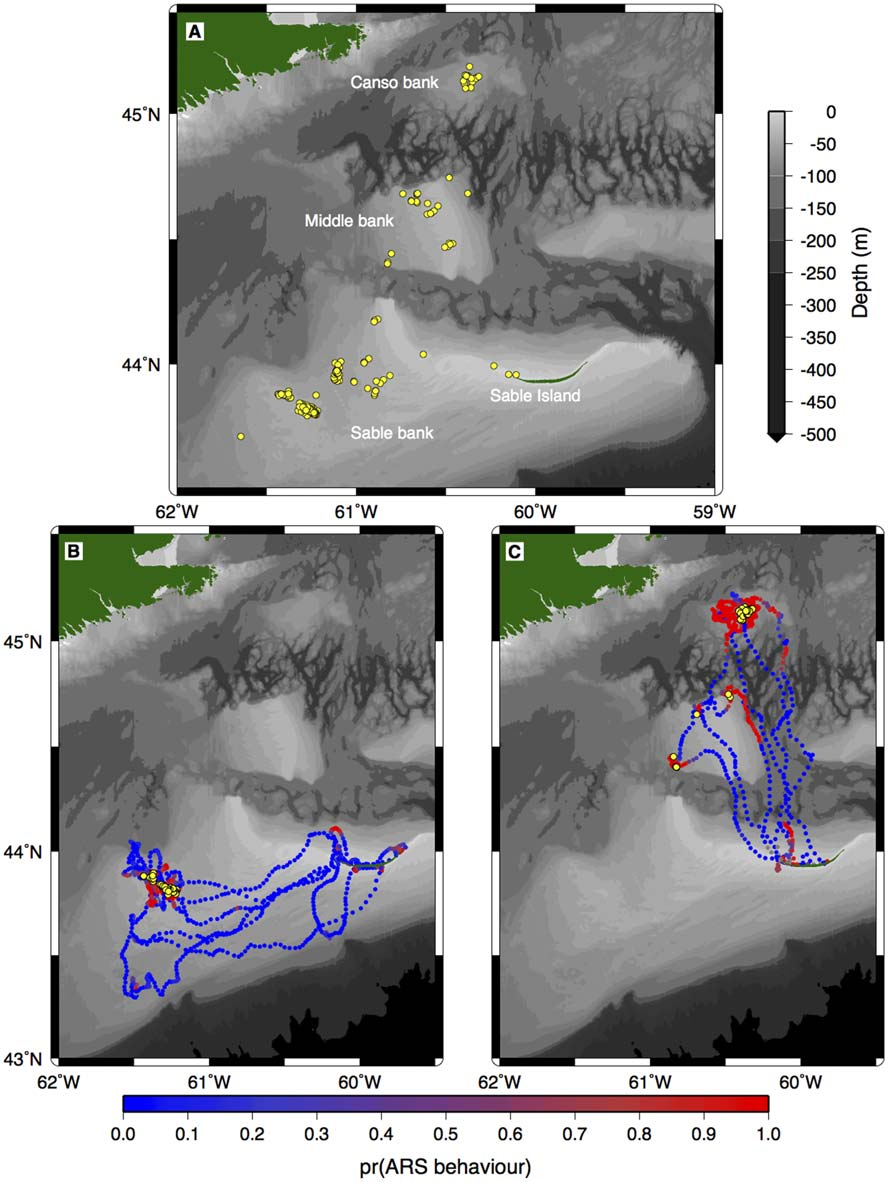
Location of associations between grey seals for A) all seals, B) seal 98430 and C) seal 98428 (*n* = 14). Figures B) and C) are examples to illustrate the correlation between location of associations, bathymetry and seal behaviour. See Fig. 1 for the pr(ARS) and depth scale legend.

**Figure 4:**
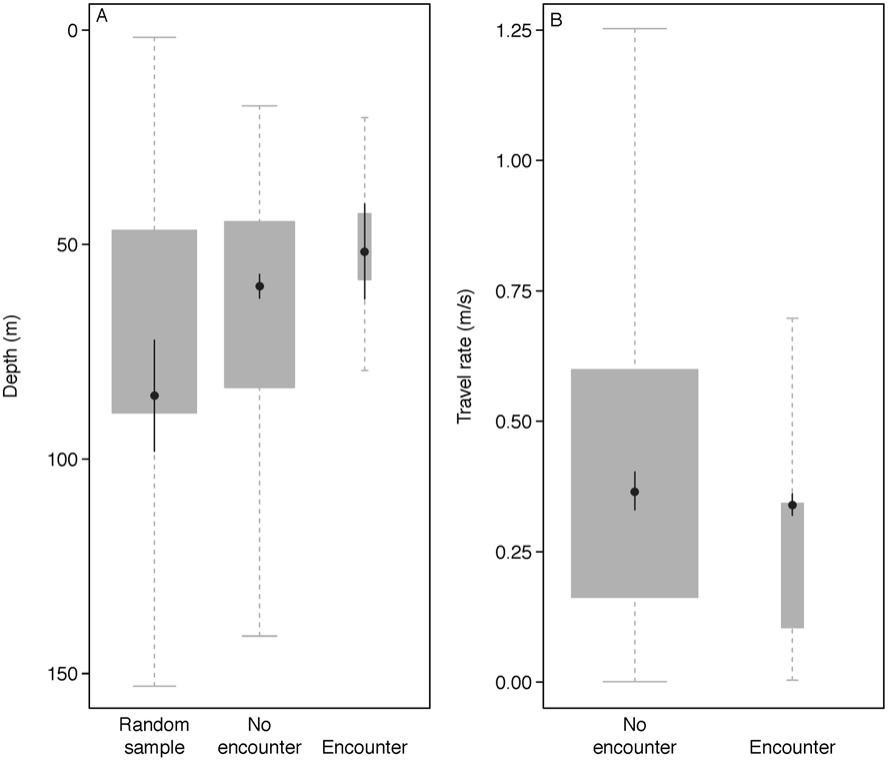
Box plots showing the distribution of A) all depths (m) and B) all travel rates (ms^−1^) according to the occurrence of associations between grey seals. A also shows the distribution of depths from 5000 randomly selected points taken from 2D kernel density plots generated from each seal track. Plus sympbols (+) are the median depth/speed for each seal, filled circles and error bars are the GLMM-estimated mean depth/speed across all seals +/−1 SE.

Seals made repeated trips from Sable Island to shallow offshore banks on the Eastern Scotian Shelf (Fig. 1B). Results from the hidden Markov model showed that seals were less likely to engage in ARS behaviour when travelling to and from Sable Island, whereas most ARS behaviour tended to occur when seals were above the offshore shallow banks (Fig. 1B). Associations were clustered above these offshore banks and during periods when seals were moving slowly (Fig. 3A to C). The generalized linear mixed-effects model results suggest that habitat used by seals was significantly shallower (no association = 74.8±89.7 m, association = 50.9±13.6 m; mean±1 sd) than depths at randomly sampled locations (85.8 m±132.3 m; mean±1 sd), regardless of whether or not the seals were associated with one another (Table 2, Fig. 4A). Seals also travelled at a slightly but significantly slower rate when associating with other seals (no association = 0.41±0.31 ms^−1^, association = 0.26±0.21 ms^−1^; mean±1 sd; Table 3, Fig. 4B).

**Table 2:** Parameter estimates from a Generalized linear mixed-effects model to estimate the differences in bottom depth (m) between a randomly sampled set of bottom depths (see methods for sampling details) and bottom depths at locations of VMT-tagged seals that were or were not associated with other VMT-tagged seals.

*Parameter*	*Depth (m)*	*SE*	*t*-value	*p-value*
Random	85.24	6.60	12.91	<0.0001
No Association	−25.54	1.42	−18.04	<0.0001
Association	−33.62	5.64	−5.96	<0.0001

Individual seals were treated as a random effect and bottom depths encountered by each seal were modelled as an AR(1) process (autocorrelation parameter estimate, *φ* = 0.274). Among-seal SD in bottom depth was 22.61 m, with a residual SD of 107.76 m.

**Table 3:** Parameter estimates from a Generalized linear mixed-effects model to estimate the differences in travel rate (ms^−1^) between VMT-tagged seals that were or were not associated with other VMT-tagged seals.

*Parameter*	*Travel rate (ms^−1^)*	*SE*	*t*-value	*p-value*
No Association	0.27	0.05	5.38	<0.0001
Association	−0.07	0.03	−2.31	0.021

Individual seals were treated as a random effect and travel rates of each seal were modelled as an AR(1) process (autocorrelation parameter estimate, *φ* = 0.789). Among-seal SD in travel rate was 0.171 ms^−1^, with a residual SD of 0.928 ms^−1^.

**Figure 5:**
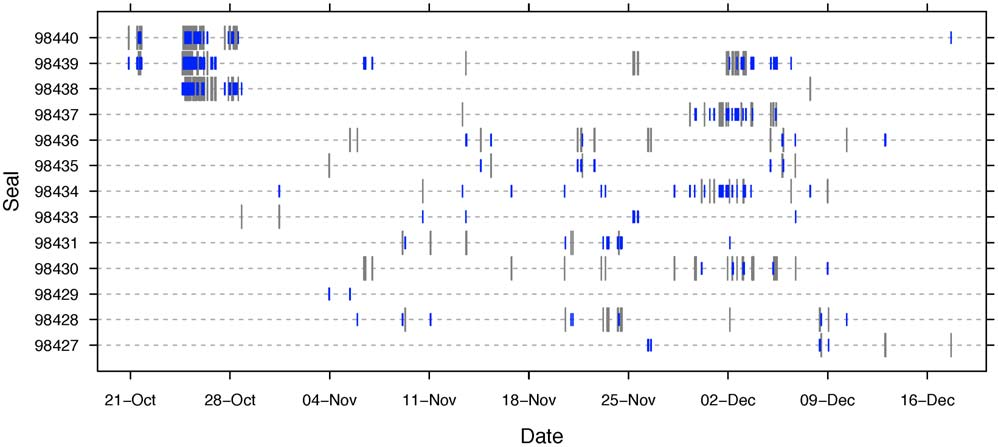
A time-series plot showing the occurrence of associations between grey seals (*n* = 13). Two-way associations (i.e., both VMTs collected detections during the association) are shown by the grey lines (receiver 1) with a matching blue line (receiver 2). The occurrence of one-way associations (i.e. only one VMT collected detections) are shown by the grey lines (receiver 1) with no matching blue line (receiver 2).

There was no clear temporal pattern of associations among individuals (Fig. 5). Three female seals exhibited a high rate of associations about a week after capture toward the end of October. However, most associations occurred in late November and early December suggesting an increase in the rate of associations just prior to returning to Sable Island for reproduction.

## Discussion

This study has shown that acoustic transceivers coupled with archival GPS telemetry can be used to study the spatial and temporal patterns of at-sea associations of grey seals. Together with demographic and environmental covariates, these data can be used to test hypotheses about the nature of those associations. For example, our preliminary data indicate a strong correlation between bathymetry, behaviour (i.e., travel rate) and geographical location suggesting seal-seal associations occur mainly above shallow offshore banks where seals are foraging. Previously, mobile acoustic transceivers have been used to examine encounters between Galapagos sharks [15] and between northern elephant seals and other acoustically tagged species during the seal's northeast Pacific migration [14]. While these two studies demonstrated the applicability of the VMT on different marine species, they were unable to address relevant biological questions due either to missing complementary positional data or low sample sizes. The current study is the first to use this novel mobile acoustic technology to address biological questions. From these studies, it is clear that mobile transceivers coupled with location data have broad application to other large marine predators (e.g., other pinnipeds, cetaceans, sharks, turtles).

Given the spatial and temporal autocorrelation inherent within these data, a statistical analysis employing association and network methodologies [39] will be required to address the nature and proximate causes of these associations. For example, without such analyses, it is difficult to interpret the multiple occurrences of associations that occurred among three individuals during the end of October. However, such an analysis will require a larger sample of individuals and associations and, therefore, is beyond the scope of this paper.

Given that ARGOS and, increasingly Fastloc™ GPS telemetry are widely used to study at-sea behaviour in pinnipeds and other marine predators, one might argue that associations could be inferred from positional data alone. However, locations derived from Service ARGOS occur at irregular times and are of varying accuracy thus limiting their usefulness for examining at-sea associations, although substantial efforts have been made to overcome these limitations [6]. Fastloc™ GPS provides more frequent and accurate locations, however without the associated VMT data the temporal and spatial scale of inferred associations will be largely governed by the diving behaviour of the study species [35]. For example, northern elephant seals average about 3 dives/h and spend ∼3 min at the surface between dives [5], thus one would expect only one or two GPS positions within each hour [35]. By comparison, female northern fur seals (*Callorhinus ursinus*) may average between 6 and 25 dives/h, and between 1.5 and 13 min at the surface, depending on whether they are foraging epipelagically or benthically [40]. Thus, with more time spent at the surface one would expect a greater number of GPS positions. However, a recent study using GPS telemetry to evaluate satellite track data in female northern fur seals, found that one may only expect ∼32 GPS locations per day, less than 2/h [41]. In both cases, the value added of VMT data is the independent verification, by receipt of an acoustic detection, that individuals were within ∼300 m or less of one another. The performance of the VMT, in terms of range of detection, requires further testing to determine how the behaviour of the predator and oceanographic (e.g. salinity, depth and temperature) and environmental (e.g., wave and wind noise) variables influence performance.

Although beyond the focus of this paper, we also expect the use of the VMT and positional data to become an important tool for studying predator-prey interactions. Marine mammals and other upper-trophic level predators (e.g. sharks, tuna, halibut) equipped with a VMT would be capable of detecting prey that have been equipped (usually implanted) with a Vemco 69 kHz coded acoustic transmitter. An inspection of the pattern of detections and duration of associations should allow one to infer the nature of the interaction, i.e., whether predation occurred or not.

There are several inferences that can be drawn from these preliminary data on the spatial pattern of associations among seals. The first is that seals associate with one another most frequently while foraging on offshore banks. This conclusion is based on the fact that seal-seal associations were clustered on offshore banks at depths ∼50 m, seals typically exhibited slow travel speeds during their time in those areas and the median distance between detections was 150 m. In the fall, grey seals of both sexes exhibit increased foraging effort, as judged by their diving behaviour and mass gain [42], [43], in preparation for the energetic demands of reproduction [42], [44]. Results from fitting a behavioural switching model to satellite tracks of adults over a ten-year period showed that during this intensified foraging period grey seals demonstrated a clear preference for shallow offshore banks on the Scotian Shelf, and exhibited area restricted searching typical of foraging [26]. The second inference is that seals appear to arrive at these foraging areas independently as there are few seal-seal associations between these areas and Sable Island. This suggests either that associations that occur during bouts of foraging are not temporally stable or that the VMT is inefficient at detecting acoustic signals while the seal is moving at speed due to higher background noise.

There are two non-mutually exclusive hypotheses for the occurrence of associations among seals while foraging. The first is based on the likelihood that seals from Sable Island associate with each other simply because they are using a favoured habitat and there is no interaction occurring between or benefit received from either participant of the association. The grey seal population in the northwest Atlantic is large (∼350,000 in 2010) and growing with most of the population associated with Sable Island (about 85% of pups born) [17], [45]. Given that the fall is the most intensive period of foraging for grey seals, and that individuals show a preference for foraging at offshore banks on the Scotian Shelf, it is reasonable to suggest that high seal density at a small number of favoured foraging hotspots may account for the clustered pattern of associations. Nevertheless, it seems remarkable that among the tens of thousands of seals present on Sable Island during the fall when the 15 seals were captured for this study, and that the mean distance between successive capture locations was 8.4 km, 14 seals would associate with one another and on multiple occasions up to 140 km from Sable Island.

The second hypothesis would suggest the existence of a social structure while at sea. Interactions occur because individuals receive greater benefit from being part of a group than being alone, including a reduction in predation risk, efficient location of prey resources, greater foraging efficiency and access to mates [46], [47]. The social structure of grey seals during the breeding season has been studied since the early 1900s and continues to this day (e.g., [48], [49]), thus much is understood. In contrast, during the non-breeding foraging period grey seals are far less accessible and, without evidence, are assumed to be solitary foragers. There are however several benefits that might drive the evolution of a non-breeding social structure. It is clear from this and previous studies that grey seals during the fall show preference for offshore banks, likely due to the presence of important prey. However, offshore banks are open simple habitats that offer a large mammal such as a grey seal few places to hide from predators, e.g., large sharks and killer whales (*Orcinus orca*) [50]. Historically, offshore banks may have been characterised as areas of high predation risk. Many studies have shown that prey alter their behaviour when exposed to a risk of predation [51], [52]. Thus, when foraging in areas where prey abundance and predation risk are high, the benefits of foraging in a group to reduce per-capita predation risk may offset the greater competition for the prey resource. Indeed, due to the high energetic demands of the approaching breeding season, both females and males may have little choice other than foraging in areas offering high quality prey despite a high predation risk. Further understanding of the foraging ecology of grey seals may be realized not only through exploring VMT seal-seal associations using more powerful tools such as network analysis and spatial point pattern analysis [53], but also through tagging likely predator and prey species with acoustic transmitters and examining seal-predator and -prey interactions [15], [54].

## Supporting Information

Figure S1 **Histogram of time between seal-seal detections (min) for grey seals on the eastern Scotian Shelf.**
(TIF)

Figure S2 **A plot of the randomly sampled locations at which bottom depth was measured (black dots) and seal locations (blue dots) for grey seals on the eastern Scotian Shelf.**
(TIF)
