# Supplementary figures and images for: Animal-Borne Acoustic Transceivers Reveal Patterns of at-Sea Associations in an Upper-Trophic Level Predator
Source: PLoS One. 2012 Nov 14;7(11):e48962. doi: 10.1371/journal.pone.0048962 (PMC3498375; doi:10.1371/journal.pone.0048962)

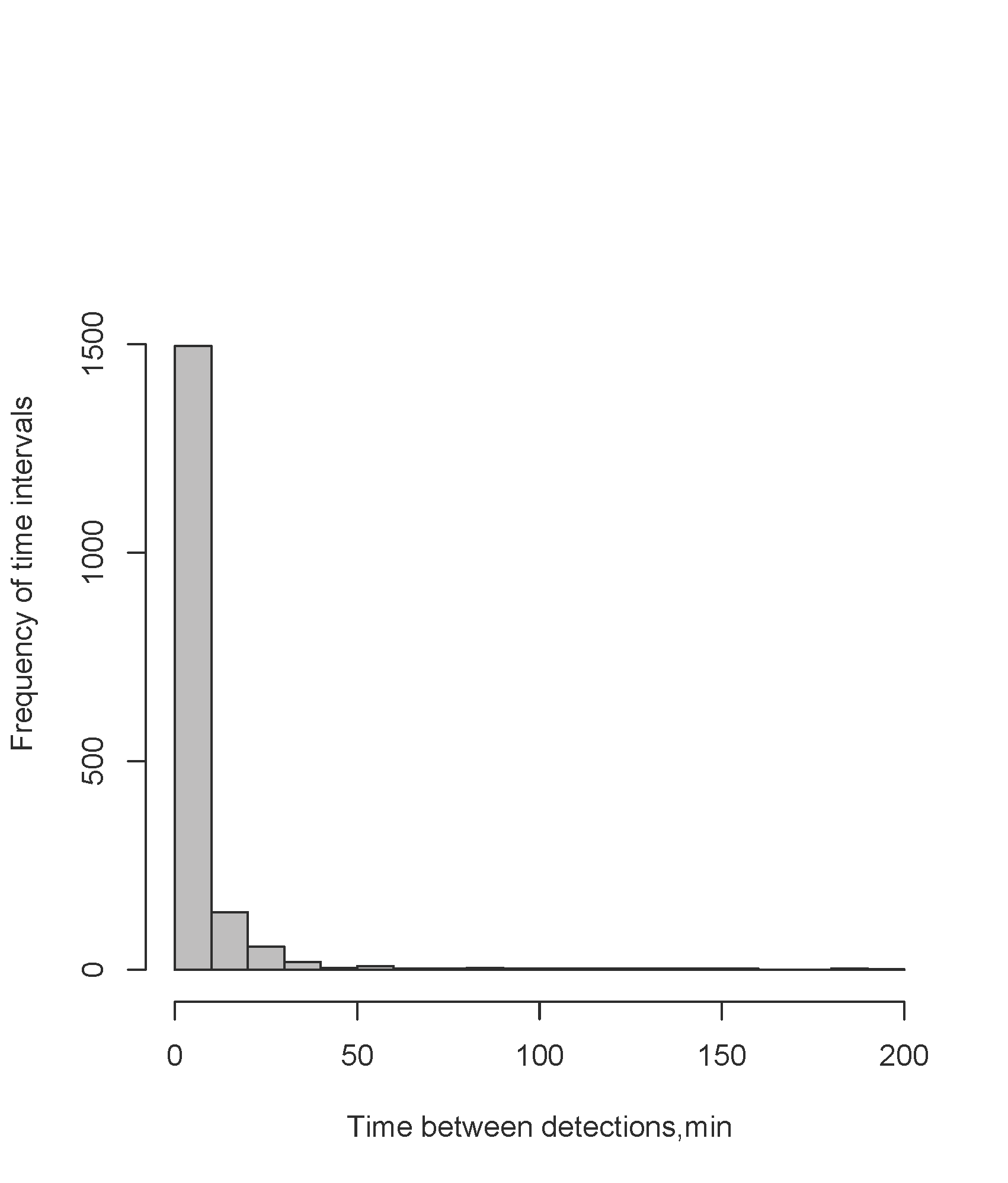

Supplement: Figure S1 — Histogram of time between seal-seal detections (min) for grey seals on the eastern Scotian Shelf. (TIF) [file pone.0048962.s001.tif]

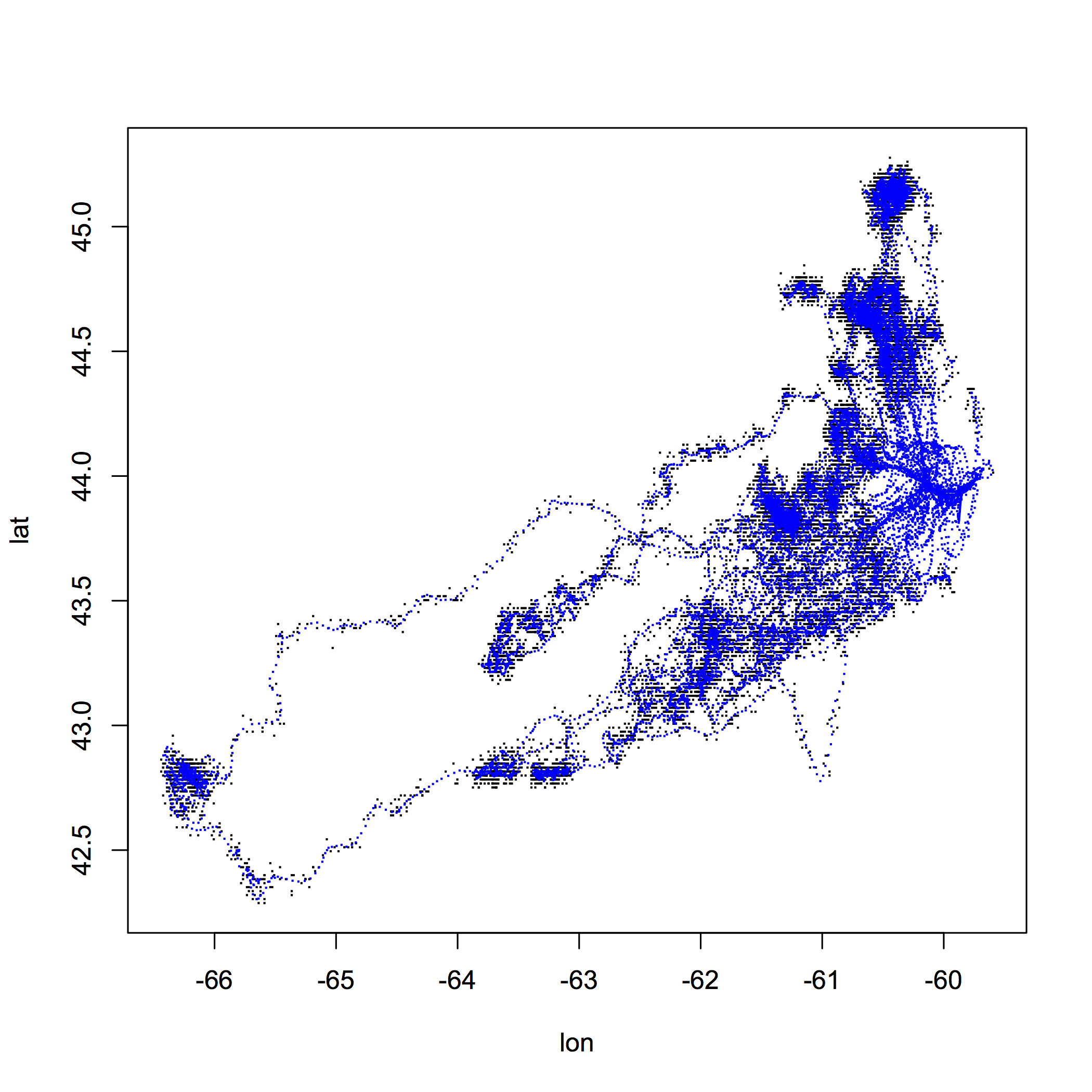

Supplement: Figure S2 — A plot of the randomly sampled locations at which bottom depth was measured (black dots) and seal locations (blue dots) for grey seals on the eastern Scotian Shelf. (TIF) [file pone.0048962.s002.tif]
